# Biomarker discovery for practice of precision medicine in hypopharyngeal cancer: a theranostic study on response prediction of the key therapeutic agents

**DOI:** 10.1186/s12885-022-09853-1

**Published:** 2022-07-16

**Authors:** Yumiko Kawata-Shimamura, Hidetaka Eguchi, Reika Kawabata-Iwakawa, Mitsuhiko Nakahira, Yasushi Okazaki, Tetsuya Yoda, Reidar Grénman, Masashi Sugasawa, Masahiko Nishiyama

**Affiliations:** 1 Department of Head and Neck Surgery, Saitama Medical University International Medical Center, 1397-1 Yamane, Hidaka, Saitama, 350-1298 Japan; 2 Research Center for Genomic Medicine, Saitama Medical University, 1397-1 Yamane, Hidaka, Saitama, 350-1298 Japan; 3 Department of Oral Surgery, Saitama Medical University, 38 Morohongo, Moroyama-machi, Iruma-gun, Saitama, 350-0495 Japan; 4 Intractable Disease Research Center, Juntendo University, 2-1-1 Hongo, Bunkyo-ku, Tokyo, 113-0033 Japan; 5 Division of Integrated Oncology Research, Gunma University Initiative for Advanced Research, 3-39-22 Showa-machi, Maebashi, Gunma 371-8511 Japan; 6 Department of Maxillofacial Surgery, Graduate School of Medical and Dental Sciences, Tokyo Medical and Dental University, 1-5-45 Yushima, Bunkyo-ku, Tokyo, 113-8549 Japan; 7 Department of Otorhinolaryngology-Head and Neck Surgery, University of Turku and Turku University Hospital, PO Box 52, 20521 Turku, Finland; 8 Gunma University, 3-39-22 Showa-machi, Maebashi, Gunma 371-8511 Japan; 9 Higashi Sapporo Hospital, 7-35, 3-3 Higashi-Sapporo, Shiroishi-ku, Sapporo, 003-8585 Japan

**Keywords:** Predictive biomarker of response, Hypopharyngeal cancer, Drug therapy, Precision medicine, Molecular target

## Abstract

**Background:**

Hypopharyngeal cancer is a relatively rare malignancy with poor prognosis. Current chemotherapeutic algorithm is still far from personalized medicine, and the identification of the truly active therapeutic biomarkers and/or targets is eagerly awaited.

**Methods:**

Venturing to focus on the conventional key chemotherapeutic drugs, we identified the most correlative genes (and/or proteins) with cellular sensitivity to docetaxel (TXT), cisplatin (CDDP) and 5-fluorouracil (5-FU) in the expression levels, through 3 steps approach: genome-wide screening, confirmation study on the quantified expression levels, and knock-down and transfection analyses of the candidates. The probable action pathways of selected genes were examined by Ingenuity Pathway Analysis using a large-scale database, The Cancer Genome Atlas.

**Results:**

The first genome-wide screening study derived 16 highly correlative genes with cellular drug sensitivity in 15 cell lines (|R| > 0.8, *P* < 0.01 for CDDP and 5-FU; |R| > 0.5, *P* < 0.05 for TXT). Among 10 genes the observed correlations were confirmed in the quantified gene expression levels, and finally knock-down and transfection analyses provided 4 molecules as the most potent predictive markers-AGR2 (anterior gradient 2 homolog gene), and PDE4D (phosphodiesterase 4D, cAMP-specific gene) for TXT; NINJ2 (nerve Injury-induced protein 2); CDC25B (cell division cycle 25 homolog B gene) for 5-FU- in both gene and protein expression levels. Overexpression of AGR2, PDE4D signified worse response to TXT, and the repressed expression sensitized TXT activity. Contrary to the findings, in the other 2 molecules, NINJ2 and CDC25, there observed opposite relationship to cellular drug response to the relevant drugs. IPA raised the potential that each selected molecule functionally interacts with main action pathway (and/or targets) of the relevant drug such as tubulin β chain genes for TXT, DNA replication pathway for CDDP, and DNA synthesis pathway and thymidylate synthetase gene for 5-FU.

**Conclusion:**

We newly propose 4 molecules -AGR2, PDE4D,NINJ2 and CDC25B) as the powerful exploratory markers for prediction of cellular response to 3 key chemotherapeutic drugs in hypopharyngeal cancers and also suggest their potentials to be the therapeutic targets, which could contribute to the development of precision medicine of the essential chemotherapy in hypopharyngeal patients. (339 words).

**Supplementary Information:**

The online version contains supplementary material available at 10.1186/s12885-022-09853-1.

## Background

Hypopharyngeal cancer is an uncommon type of head and neck cancer with significantly poor prognosis [1–3]. The malignancy accounts for approximately 0.4% of all new cancer cases and of death worldwide, and the 5-year survival rate stays at only 30–35%. The anatomical characteristics make it difficult for patients to be aware of the initial symptom. Hence, majority of patients are diagnosed at advanced stage after the appearances of dysphagia, dyspnea, and/or the metastases to cervical lymph nodes. Moreover, the cancer is aggressive, invasive and heterogeneous, which makes the disease highly intractable [4–6]. Despite of the remarkable progress in the therapeutic modalities, hypopharyngeal cancer is still a challenging disease to treat [7–10].

The treatment of the patients includes surgery, radiotherapy, chemotherapy, and immunotherapy, and multimodal approaches such as surgery with chemo-radiotherapy has been widely applied [4–12]. Surgical treatment provides an opportunity for cure, and the procedures have been increasingly progressed [13]. Not a few cases, however, remain to require resection of larynx near hypopharynx, which is often associated with poor functional results including vocal, swallowing, and chewing disorders [13, 14]. Intensive chemotherapy and/or irradiation offers acceptable oncologic and functional outcomes, but consecutively the therapy-associated drug resistance and recurrence were frequently observed [5–12]. The advent of molecular target agents and immunotherapy have been innovating on the classical treatment algorithms in a variety of cancers**,** but the therapeutic impact is limited in hypopharyngeal cancer [15–17].

Further improvements in prognosis and QOL in the malignancy are dependent upon the discovery of the disease-specific molecules or genomic alterations that can be used as therapeutic targets and powerful predictive biomarkers of individual response to the therapy, which could lead to precision medicine with truly active drug targets [6, 9, 15–17]. Omics research has been increasing the understanding of molecular basis underlying the disease [18–20]. Even so, the great heterogeneity of both clinical and biological characteristics, including the status with or without human papillomavirus infection, becomes a big obstacle to elucidate the cancer-specific mechanisms and the critical action molecules in hypopharyngeal cancer [1, 6, 9, 10].

At present, individual response to the current chemotherapy, therefore, is of key importance in the preservation of pharyngeal functions, deglutition, and phonation, which determines quality of life (QOL) of the patients. With the accumulated evidence of the therapeutic advantage, Docetaxel (TXT), cis-Platinum (CDDP), and 5-fluorouracil (5-FU) still play a central role in the drug treatment [21–23]. Even for the response prediction of these cytotoxic agents, however, any definitive predictive biomarkers have not been identified**,** despite of the extensive studies [24–26].

In this study, focusing on these 3 key drugs, we explored their putative sensitivity- and/or resistance-determinant genes, as the first solid step to the goal. This would allow selection of a most active regimen for individual patient, as well as prevention of an unnecessary treatment that could contribute the preservation of pharyngeal functions. The identification of the putative resistant determinants also might possibly unlock the future for disease-specific drug discovery in hypopharyngeal cancers.

## Methods

### Drugs and chemicals

CDDP was purchased from Sigma-Aldrich (St.Luis, US). TXT was obtained from Toronto Research Chemicals (Toronto, Canada) and 5-FU was received from Wako Pure Chemical Industries (Osaka, Japan). All other chemicals were purchased from Nacalai Tesque (Kyoto, Japan), Wako Pure Chemical Industries, or Sigma-Aldrich.

### Cell lines

Since the number of obtainable hypopharyngeal squamous cell carcinoma (SCC) cell lines were limited, a total of 15 pharyngeal SCC cell lines were used in this study: They were 9 hypopharyngeal SCC cell lines (UT-SCC-26A, −26B, − 62, − 66, − 70, − 89, − 94, BICR6, and HPC-921Y) and 6 other pharyngeal SCC cell lines including 2 mesopharynx (MPC-881 T and -882Y), 2 oropharynx (UT-SCC-4, and UMB-ACC-745), and 2 pharynx SCC cells (FaDu and Detroit 562). A series of UT-SCC cell lines were kindly provided by Professor Reidar Grénman, Department of Otorhinolaryngology - Head and Neck Surgery, University of Turku and Turku University Hospital, Turku, Finland [27–29]. BICR 6 was purchased from European Collection of Cell Cultures (ECACC). HPC-921Y, MPC-881 T, and MPC-882Y cells were kindly provided by Dr. S. Yanoma, Yokohama City University, Yokohama, Japan, and UMB-SCC-745 was gratefully offered by Dr. Robert Mandic, Department of Otolaryngology, Head & Neck Surgery, University Hospital Giessen and Marburg, Giessen and Marburg, Germany [30]. FaDu and Detroit 562 were supplied from American Type Culture Collection (ATCC) [31]. Cells were cultured in recommended medium containing 10% heat-inactivated fetal bovine serum (FBS; BioWhittaker, Verviers, Belgium) and 50 μg/mL kanamycin-sulfate. All cultured cells were incubated at 37 °C in a humidified atmosphere of 5% CO_2_ and maintained in continuous exponential growth by passaging. Mycoplasma test and short-tandem repeat profiling were performed in regular basis from the first culture of the cells to verify the cells to be the same as the cells registered.

### Cytotoxic assay

Drug-induced cytotoxicity was evaluated by conventional MTT [3-(4,5-dimethylthiazol-2-yl)-2,5-diphenyltetrazolium bromide] dye reduction assay as previously reported [25]. Briefly, 4 × 10^3^/well cells were seeded in 96-microwell plates (Cornig, NY, US) with complete medium containing 10% FBS (fetal bovine serum). After 24 h of incubation, the medium was replaced and cells were exposed to the indicated drug concentrations for 72 h, after which 10 μl of 0.4% MTT reagent and 0.1 M sodium succinate were added to each well. After 2 h of incubation, 150 μl of DMSO was added to dissolve the purple formazan precipitate. The formazan dye was measured spectrophotometrically (570–650 nm) using MAXlineTM microplate reader (Molecular Devices Corp., Sunnyvale, US). The cytotoxic effect of each treatment was assessed by IC_50_ value (inhibitory drug concentration of 50% cell growth: drug concentration of 50% optical density of control).

### Extraction of RNA

For gene expression analysis, total RNA of cell pellets was prepared from each cell using NucleoSpin RNAIIPurfication Kit (Macherey-Nagel, Düren, Germany). The quality of RNA was evaluated using Agilent Technologies 2100 Bioanalyzer (Agilent, Santa Clara, US).

### Comprehensive gene expression analysis using microarray

After reverse-transcription from total RNA, labeled cRNA was produced using Quick Amp Labeling Kit (Agilent). We measured gene expression levels of total RNA and non-cording RNA using SurePrint G3 Human GE micro array analysis 8x60K (Agilent Technologies Japan, Ltd., Tokyo, Japan). Scanned image data was digitized using Agilent Feature Extraction software (Agilent Technologies Japan, Ltd.). The signal intensity of gene expression was standardized by the quantile normalization method recommended by Agilent company using analysis environment language R (http://www.r-project.org/).

### Quantitative gene expression analysis

The gene expression levels were quantified by real-time PCR. Total RNA extracted from each cell was reverse-transcribed using ReverTra Ace® qPCR RT Kit (TOYOBO Co., Osaka, Japan), and then the cDNA was subjected to real-time reverse transcription-PCR (RT-PCR), which was conducted using specific primer and probe sets (Invitrogen, Carlsbad, US) designed by Universal ProbeLibrary Assay Design Center (https://qpcr.probefinder.com/organism.jsp) (Roche Dianostics, Basel, Swizerland), UPL probe (Roche Dianostics), amplification reagents [qPCR QuickGoldStar Mastermix Plus (Nipongene, Tokyo, Japan) or ABsolute QPCR Mix (Thermo Fisher Scientific, Yokohama, Japan)], and LightCycler480 (Roche Dianostics). Used primers and probes were showed in Supplementary File 1. Real-time PCR analyses for *ACTB* (action beta gene) and *GAPDH* (glyceraldehyde-3-phosphate dehydrogenase gene) were performed using Endogenous Control (VIC® / MGB Probe, Primer Limited) (Invitrogen). Each reaction was carried out in triplicate and averaged, and relative gene expression level was calculated as a ratio to mean expression level of *ACTB*, *GAPDH*, and *HPRT1* (hypoxanthine phosphoribosyltransferase 1 gene).

### Knockdown analyses of *AGR2* and *NINJ2* using siRNA

Specific siRNAs [(Stealth RNAi (Invitrogen)] for *AGR2* (anterior gradient 2 homolog gene; HSS116220, HSS173724, and HSS116219) and *NINJ2* (nerve Injury-induced protein 2 gene; HSS107192, HSS181530, and HSS107191), and negative control siRNA (Stealth RNAi Negative control siRNA; Hi GC Duplex, Med GC Duplex, and Low GC Duplex) were used for knockdown experiments. We selected negative control siRNAs which were appropriate for contents of GC in each RNAi. Cell lines were transfected with siRNAs by electroporation using device CUY21Pro-Vitro (Nepagene, Ichikawa, Japan). After incubation, total RNA was extracted and purified from each cell. Then the gene expression levels were quantified by real-time RT-PCR, and we validated knockdown efficiencies.

Condition of electroporation was optimized according to manufacture ‘s protocol. Concretely, cell lines were transfected with pCMV-EGFP plasmid (Nepagene). We validated transfection efficiencies and viable cell rates using fluorescence microscope images and BD FACS Calibur flow cytometer (Becton, Dickinson and Company, Franklin Lakaes, US). Used condition of electroporation were here; poring pulse (Pp) was 275 V, pulse duration was 0.5 ms, pulse interval was 50 ms, driving pulse (Pd) was 20 V, pulse duration was 50 ms, pulse interval was 50 ms, and pulse frequency was 10.

### Construction of plasmid

After reverse-transcription from total RNA from cell lines, PCR reaction was carried out using KOD-Plus or KOD FX (TOYOBO) as a DNA polymerase, according to the manufacture’s protocol. Coding region of *AGR2, PDE4D* (phosphodiesterase 4D, cAMP-specific gene)*, RAB15* (member RAS onocogene family gene)*, CDC25B* (cell division cycle 25 homolog B gene) and *RCAN3* (RCAN family member 3 gene) were amplified using specific primers (See Supplementary File 2). Then they were digested with XbaIand ligated into cloning sites of pRc/CMV-HA digested with Eco72I and XbaI. They were transformed into competent E.coli DH5α(TOYOBO) using Ligation high Ver.2 (TOYOBO) according to the manufacture ‘s manual. All constructs were confirmed by DNA sequencing using BigDye ○R Terminator 3.1 Cycle Sequencing Kit and 3130 Genetic Analyzer (Life Technologies, Tokyo, Japan).

### Molecular network and functional analyses

To examine the molecular network and biological function of target genes, IPA (Ingenuity Pathway Analysis, QIAGEN Redwood City; Content version 68,752,261, Release Date 2021-09-06) was used as follows: 1) Add molecules on “my pathway”. 2) Connect direct and/or indirect relationship among molecules using “Path Explorer” with default setting excluding microRNA database. 3) Overlay selected “Disease & Function” with default setting.

### Transfection and selection of stable transformants

Plasmids expressing each gene were linearized by a single cut with a restriction enzyme. They were transfected into hypopharyngeal cell lines by electroporation. Transfected cells were cultured in recommended medium with 10% heat-inactivated FBS containing 300–500 μg/mL of G418 from 24 hours after transfection for 1–2 month to select stable transfected clones. We measured gene expression levels of mRNA in transfected cells using real time PCR and subsequently performed Western blot analyses to confirm the results also in the protein expression levels (Supplementary Information File 1_Western Blot raw Data). The immunoblotting data were quantified by using image analysis system, Image Quant LAS4000 (GE Healthcare Japan Corporation, Tokyo, Japan).

### Statistical analyses

Statistical analysis was performed using R software and JMP^Ⓡ^ (JMP Japan, Tokyo, Japan). The correlation between drug sensitivity and gene expression value was analyzed using linear regression analysis.

## Results

### Screening of probable genes associated with sensitivity to key therapeutic drugs

Using data sets obtained by microarray gene expression analysis and cytotoxic assay (Table 1) in 15 pharyngeal cell lines, we first sorted out genes which were correlative in expression level with cellular sensitivity to 3 key therapeutic drugs, TXT, CDDP and 5-FU (See Supplementary Dataset Files 1, 2, 3). The linear regression analyses demonstrated that 4 and 8 genes were closely correlated with cellular sensitivity to CDDP and 5-FU, respectively (|R| > 0.80, *P* < 0.01). However, for TXT, we couldn’t find any correlative gene other than *AGR2*. To avoid losing potent marker genes, we extended the screening field up to |R| > 0.50 (*P* < 0.05) and selected another 3 correlative genes, *SYNGR1* (synaptogyrin 1 gene), *PDE4D*, and *RAB15* for TXT. We finally picked a total of 16 genes in this first screening (Table 2).

**Table 1:** Cellular sensitivity to 3 key therapeutic drugs, docetaxel (TXT), cisplatin (CDDP), and 5-fluorouracil (5-FU)

Cell Line	Histology	Sensitivity to drug (IC_50_)		
		TXT (pg/ml)	CDDP (μg/ml)	5-FU (μg/ml)
UTC-SCC-26A	hypopharyngeal squamous cell carcinoma	47.9094	8.3860	0.8209
UTC-SCC-26B	hypopharyngeal squamous cell carcinoma	0.1690	0.2828	0.0557
UTC-SCC-62	hypopharyngeal squamous cell carcinoma	6.2064	1.6152	1.1576
UTC-SCC-66	hypopharyngeal squamous cell carcinoma	207.9236	1.6044	0.1212
UTC-SCC-70	hypopharyngeal squamous cell carcinoma	9191.0864	2.3567	0.2077
UTC-SCC-89	hypopharyngeal squamous cell carcinoma	0.0940	0.5494	0.0435
UTC-SCC-94	hypopharyngeal squamous cell carcinoma	0.7811	1.3697	0.0766
BICR6	hypopharyngeal squamous cell carcinoma	0.1036	0.2122	0.4808
HPC-921Y	hypopharyngeal squamous cell carcinoma	9980.4473	2.8667	1.1515
MPC-881 T	mesopharyngeal squamous cell carcinoma	0.0422	0.3488	0.0548
MPC-882Y	mesopharyngeal squamous cell carcinoma	22,607.4900	0.3596	0.9589
UTC-SCC-4	oropharyngeal squamous cell carcinoma	8898.8459	0.9443	39.8870
UMB-ACC-745	oropharyngeal squamous cell carcinoma	0.0936	0.9743	0.3083
FaDu	pharyngeal squamous cell carcinoma	3798.9334	1.0897	2.2402
Detroit562	pharyngeal squamous cell carcinoma	3.3620	1.5133	2.1239

IC_50_, inhibitory drug concentration of 50% cell growth: drug concentration of 50% optical density of control

**Table 2:** Screening of probable marker genes highly correlated with cellular sensitivity to key therapeutic drugs

Drug	Gene symbol	Gene name	Correlation between gene expression and drug sensitivity							
			Microarray analysis				RT-PCR analysis			
			R	|R|	R^2^	P	R	|R|	R^2^	P
TXT	*AGR2*	anterior gradient 2 homolog	0.7009	0.7009	0.4913	**0.0036**	0.6974	0.6974	0.4864	**0.0039**
	*SYNGR1*	synaptogyrin 1	0.6262	0.6262	0.3921	0.0125	0.4533	0.4533	0.2055	0.0897
	*PDE4D*	phosphodiesterase 4D, cAMP-specific	0.5885	0.5885	0.3463	**0.0210**	0.7462	0.7462	0.5568	**0.0014**
	*RAB15*	RAB15, member RAS oncogene family	0.5298	0.5298	0.2807	**0.0422**	0.6366	0.6366	0.4053	**0.0107**
CDDP	*NOTCH2NL*	notch 2 N-terminal like	0.7575	0.7575	0.5738	0.0011	0.0361	0.0361	0.0013	0.8984
	*KLK11*	kallikrein-related peptidase 11	−0.7526	0.7526	0.5664	**0.0012**	−0.6700	0.6700	0.4489	**0.0063**
	*NINJ2*	ninjurin 2	−0.8180	0.8180	0.6691	**0.0002**	−0.8532	0.8532	0.7280	**0.0001**
	*PTGS1*	prostaglandin-endoperoxide synthase 1	−0.8309	0.8309	0.6904	**0.0001**	−0.7722	0.7722	0.5963	**0.0007**
5-FU	*GOLGA8A*	golgin A8 family, member A	0.7857	0.7857	0.6173	0.0005	−0.1010	0.1010	0.0102	0.7202
	*JDP2*	Jun dimerization protein 2	0.7665	0.7665	0.5875	0.0009	0.4067	0.4067	0.1654	0.1325
	*STXBP1*	syntaxin binding protein 1	−0.7504	0.7504	0.5631	0.0013	−0.3824	0.3824	0.1462	0.1595
	*CDC25B*	cell division cycle 25 homolog B	−0.7528	0.7528	0.5667	**0.0012**	−0.6434	0.6434	0.4140	**0.0097**
	*PBX3*	pre-B-cell leukemia homeobox 3	−0.7599	0.7599	0.5774	**0.0010**	−0.5457	0.5457	0.2978	**0.0354**
	*SEPW1*	selenoprotein W, 1	−0.7648	0.7648	0.5849	**0.0009**	−0.7058	0.7058	0.4982	**0.0033**
	*RCAN3*	RCAN family member 3	−0.8006	0.8006	0.6410	**0.0003**	−0.6375	0.6375	0.4064	**0.0106**
	*ZNF584*	zinc finger protein 584	−0.8344	0.8344	0.6962	0.0001	0.0574	0.0574	0.0033	0.8390
	*(AGR2)*						0.5883	0.5883	0.3461	**0.0211**

These genes were then subjected to real-time PCR analysis to verify whether the observed correlations were confirmed even in the quantified expression levels. We confirmed 10 of 16 candidate genes were still highly correlative to cellular drug sensitivity: They were *AGR2*, *PDE4D* and *RAB15* for TXT; *KLK11* (kallikrein-related peptidase 11 gene), *NINJ2* and *PTGS1* (prostaglandin-endoperoxide synthase 1 gene) for CDDP; and *CDC25B*, *PBX3* (pre-B-cell leukemia homeobox 3 gene), *SEPW1* (*SELENOW*: selenoprotein W 1 gene) and *RCAN3* for 5-FU. Despite of the lack of correlation in the first screening*,* we found that *AGR2* were also correlated with cellular drug sensitivities to 5-FU in the quantified expression levels (Table 2).

Among these, *AGR2, PDE4D* and *RAB15* were positively correlated with the IC_50_ values of TXT, indicating that an increased levels of their expression caused an increase of cellular resistance to the drug. Oppositely, the expression levels the other 7 genes were negatively correlated with IC_50_ values of the relevant drug, indicating that an increase of their expressions sensitized cells to the drugs.

### Biological interactions of selected genes with drug action mechanisms

These selected 10 genes would be potent candidates of predictive biomarkers for the relevant drugs, TXT, CDDP, or 5-FU (*AGR2* was shown to be related to both TXT and 5-FU), but their clinical and/or biological roles as drug sensitivity (or resistance) determinants remain to be elucidated.

**Fig. 1:**
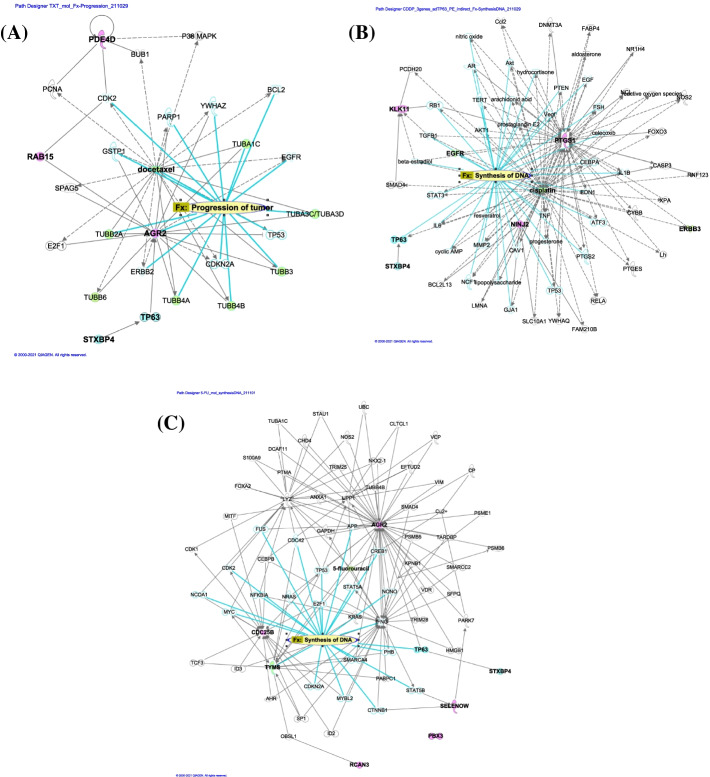
Ingenuity pathway analysis (IPA) using the knowledge database. Probable molecular networks including interrelations with syntaxin binding protein 4 (STXBP4) and canonical pathways of 10 highly correlative genes with cellular drug response were assessed by IPA, of which results were demonstrated for each target drug, docetaxel (TXT) (**A**), cis-platinum (CDDP) (**B**), and 5-fluorouracil (5-FU) (**C**)

We first attempted to analyze their possible clinical prognostic impact, using a large-scale public database, TCGA (The Cancer Genome Atlas), but none of the reliable findings were obtained due to the very limited number of data sets, genes expression data and clinical information of hypopharyngeal cancer patients. Meanwhile, ingenuity pathway analysis (IPA) using the knowledge database suggested that the selected genes might functionally interact with action target genes of the relevant drug (Fig. 1). *AGR2, PDE4D,* and *RAB15* may play some significant roles in cancer progression and functionally connect with action targets of the microtubulin disassembly inhibitor TXT, tubulin β chain genes such as *TUBB2, TUBB3, TUBB4* and *TUBB6* (Fig. 1 (A), Supplementary Dataset File 4). *KLK11*, *NINJ2* and *PTGS1* are probably involved in DNA replication pathway, which relates to main action mechanisms of CDDP, interference of DNA replication by eliciting drug-DNA cross links (Fig. 1 (B), Supplementary Dataset File 5). *CDC25B*, *PBX3*, *SEPW1* (*SELENOW*) and *RCAN3* were supposed to participate in the pathways of cell death and survival, cancer cell proliferation, and cancer organismismal injury and abnormalities, and further drug action target gene of 5-FU*, TYMS* (thymidylate synthetase gene) (Fig. 1 (C), Supplementary Dataset File 6). Likewise, *AGR2* (via KRAS and TP53) were also shown to be possibly related to *TYMS* (via obscurin like cytoskeletal adaptor 1 gene, *OBSL1*). Despite the detailed action are still unknown, each of selected 10 genes is probably involved in key action mechanisms of the relevant drug, TXT, CDDP, and/or 5-FU.

We simultaneously examined the relevance of these putative marker genes to syntaxin binding protein 4 (*STXBP4*), because we recently found that STXBP4 plays a crucial role in SCC growth through regulation of ΔNp63 (an isoform of tumor protein 63, TP63) ubiquitination and is an independent prognostic factor signifying a worse outcome in lung SCC patients [26, 32, 33]. IPA analysis indicated that there might exist some biological interactions between *STXBP4* and *AGR2* via *TP63*, but in the other 9 selected genes such interaction with *STXBP4* was not observed (Fig. 1).

### Functional significance of selected genes as predictive biomarkers and/or therapeutic targets

In the expression levels, 10 selected genes were significantly correlated with cellular response to 3 key drugs in 15 cell lines and might interact with main action pathway of the drugs, which led us to focus on these genes as the most plausible predictive marker genes in hypopharyngeal cancers. To elucidate the potential, we performed knock-down and/or transfection analysis of each gene, which revealed that expression of 4 genes except *RAB15* and *RCAN3* still closely related to the observed cellular sensitivities (or resistance) to the relevant drugs even in the variant clones and/or transfectants: They are *AGR2* and *PDE4D* for TXT, *NINJ2* for CDDP, and *CDC25B* for 5-FU.

**Fig. 2:**
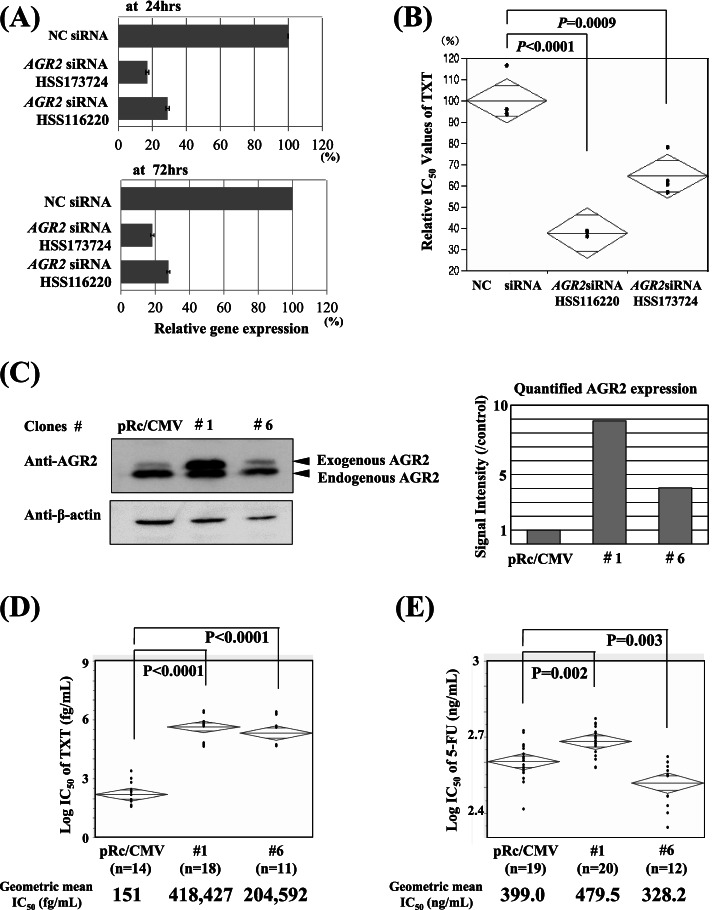
Effect of knockdown and transfection of *AGR2* on cellular TXT sensitivity. Treatment of UT-SCC-70 cells with AGR2-specific siRNAs (HSS173724 and HSS16220) significantly repressed cellular *AGR2* expression both at 24 and 72 hrs after initiation of the treatment. **A**. The repression of *AGR2* expression was associated with a decrease of IC_50_ values of TXT (docetaxl), thereby sensitizing the cells to the drug (**B**). In contrast, *AGR2* transfection into UMB-SCC-745 cells enhanced the expression both in the gene and protein expression levels (**C**), which increased IC_50_ values of TXT in the stable *AGR2* transfectants (#1 and #6). The enhanced cellular TXT resistance was well associated with the enhanced *AGR2* expression (**D**). Western blot analyses and quantification of the blots were performed for AGR2 and β-actin and the blots displayed were cropped from each transferred membrane (**C**) (See Supplemental Information File 1_Western Blot Raw Data). These expression-drug sensitivity associations were not always observed against 5-FU (5-fluorouracil), although AGR2 was suggested to be involved in also 5-FU sensitivity (**E**): *AGR2* expression was normalized with the geometric mean of *GAPDH*, *ACTB* and *HPRT1* expression and showed as relative expression to that in Negative Control (NC); Error bars represent standard errors (SE); IC_50_, inhibitory drug concentration of 50% cell growth or drug concentration of 50% optical density of control; Relative IC_50_ value of TXT (%) represents the ratio to that in control sample

Repression of *AGR2* expression induced by SiRNA treatment in UMB-SCC-745 cells caused a significant decrease of the IC_50_ value for TXT (Fig. 2 A, B), while overexpressed AGR2 yielded by the gene transfection to the cells did increase the IC_50_ value of TXT. AGR2 overexpression resulted in cellular resistance to TXT, and the repression sensitizes cells to TXT (Fig. 2 C, D). Correlation analysis on gene expression vs drug sensitivity in 15 cell lines suggested that *AGR2* was also a possible resistant predictor to 5-FU, but the expected correlation with drug sensitivity could not be observed in UMB-SCC-745 transfectants overexpressed AGR2 (Fig. 2 C, E).

**Fig. 3:**
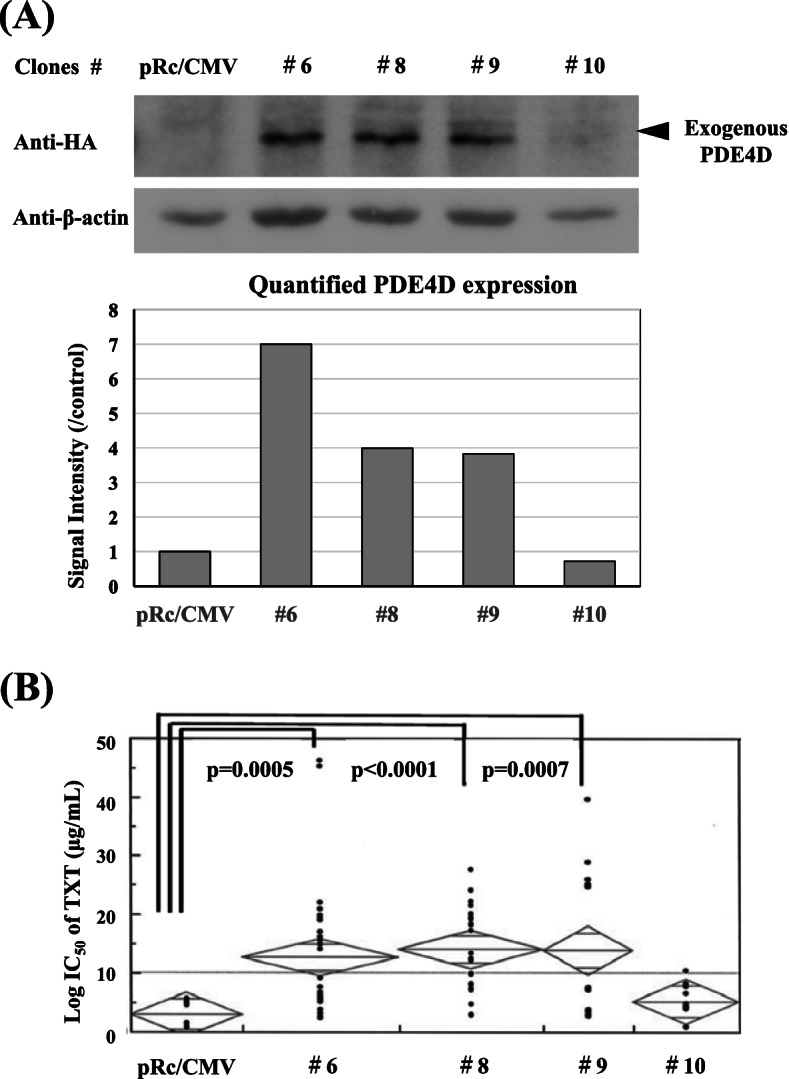
*PDE4D* transfection and cellular TXT sensitivity. As in the case of RAB15, PDE4D overexpression in BICR6 transfectants (#6, #8, #9, and #10) (**A**) caused an increase of TXT resistance in association with the enhanced expression levels (**B**). Western blot analyses and quantification assay on the blots were performed for the target protein and β-actin (**A**) (See Supplemental Information File 1_Western Blot Raw Data)

**Fig. 4:**
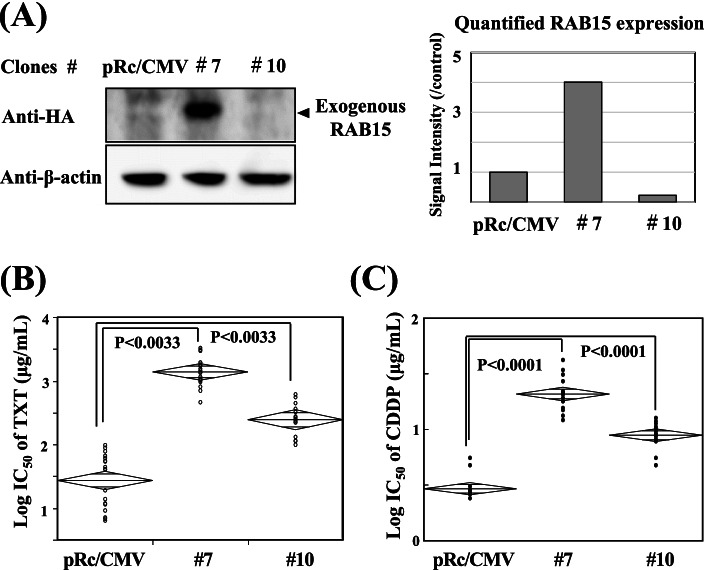
*RAB15* transfection and cellular drug sensitivity. *RAB15* transfection to BICR6 cells provided 2 stable transfectants with having different levels of RAB15 overexpression (#7 and #10)**,** which was shown as the results obtained in Western blot analyses and the following quantification assay on the blots (**A**) (See Supplemental Information File 1_Western Blot Raw Data). Although IC _50_ values of TXT in the transfectant #7 were increased in accordance with an increase of RAB15 expression**,** such expression-drug sensitivity interaction was not observed in BICR6 variant #10 (**A, B**). Unexpectedly, the enhanced expression of RAB15 led to an enhancement of cellular CDDP (cisplatin) resistance in the transfectant #7 but did not in BICR6 variant #10 in the similar manner with TXT (**A, C**)

Differing from their unsettled gene repression by the specific SiRNA treatment, gene transfection of *RAB15* and *PDE4D* to BICR6 cells provided several stable transfectants (#6, 8, 9 for *PDE4D,* #7 for *RAB15*) (Figs. 3A and 4A). We found that the enhanced protein expressions of PDE4D caused an increase of each IC_50_ value of TXT in association with an increase of the expression level as with AGR2 **(**Fig. 3 A, B), but such expected correlation was not confirmed in *RAB15* transfectants (Fig. 4 A, C). Although enhanced RAB15 in the transfectant #7 increased cellular resistance to TXT and further to CDDP, but these increased drug resistances were observed also in BICR6 variant #10 without obtaining any enhanced protein expression by *RAB15* transfection (Fig. 4A, B, C).

**Fig. 5:**
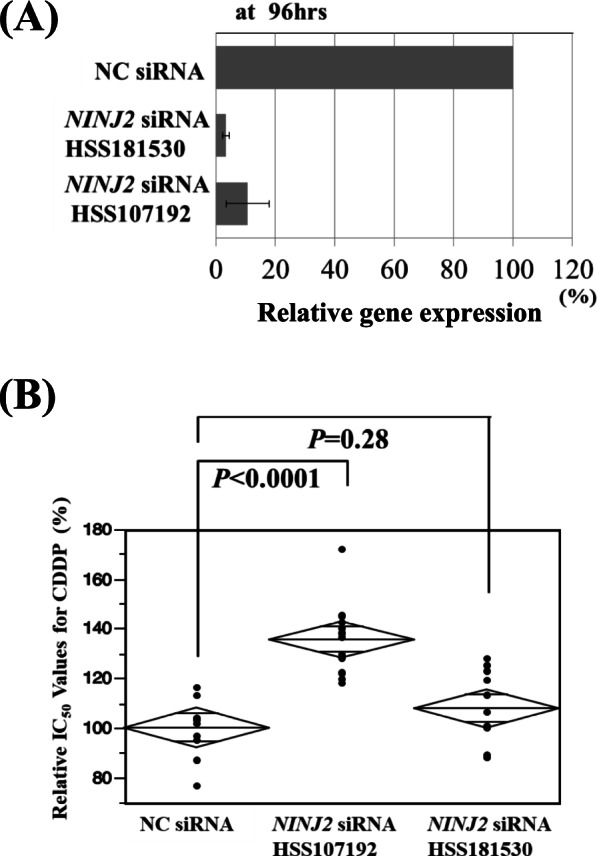
*NINJ2* knockdown and cellular CDDP sensitivity. Treatment of UT-SCC-89 cells with *NINJ2*-specific siRNAs (HSS181530 and HSS107192) induced significant repression of *NINJ2* expression even after 96 hrs from initiation of the treatment (**A**) (See Supplemental Information File 1_Western Blot Raw Data). The repression of *NINJ2* expression was associated with an increase of IC_50_ values of CDDP, thereby enhancing cellular CDDP resistance (**B**)

For CDDP, *NINJ2* expression alone was shown to be closely connected to cellular sensitivity to the drug. The repression of *NINJ2* expression by its specific SiRNA yielded a significant increase of IC_50_, thereby increasing CDDP resistance in UT-SCC-89 cells (Fig. 5). For *KLK11* and *PTGS1*, none of the definitive associations between gene expression and cellular CDDP sensitivity was confirmed in a series of both knock-down and transfection analyses performed.

**Fig. 6:**
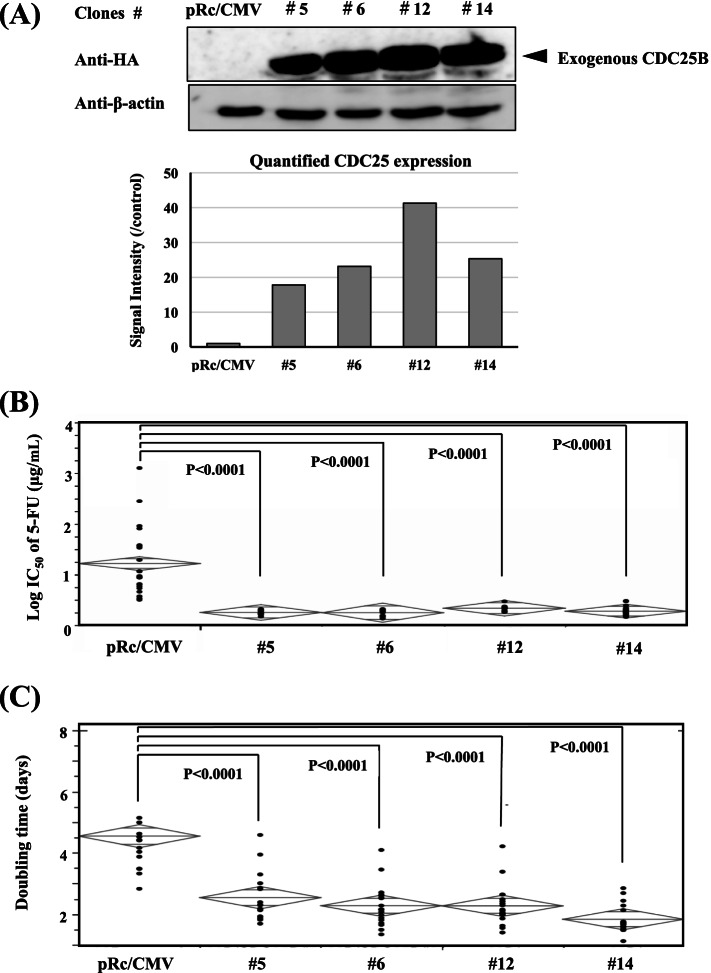
Effect of CDC25B overexpression on cellular 5-FU sensitivity and doubling time. *CDC25B* transfection to UT-SCC-26B cells provided 4 stable transfectants with having different levels of CDC25B overexpression (#5, #6, #12 and #14) (**A**). The induction of the gene expression caused a significant decrease of IC _50_ values of 5-FU in the transfectants, thus sensitizing the cells to 5-FU (**B**), and significantly shorten the doubling time of each transfectant (**C**). Western blot analyses and quantification assay on the blots were performed for the target protein and β-actin (**A**) (See Supplemental Information File 1_Western Blot Raw Data)

**Fig. 7:**
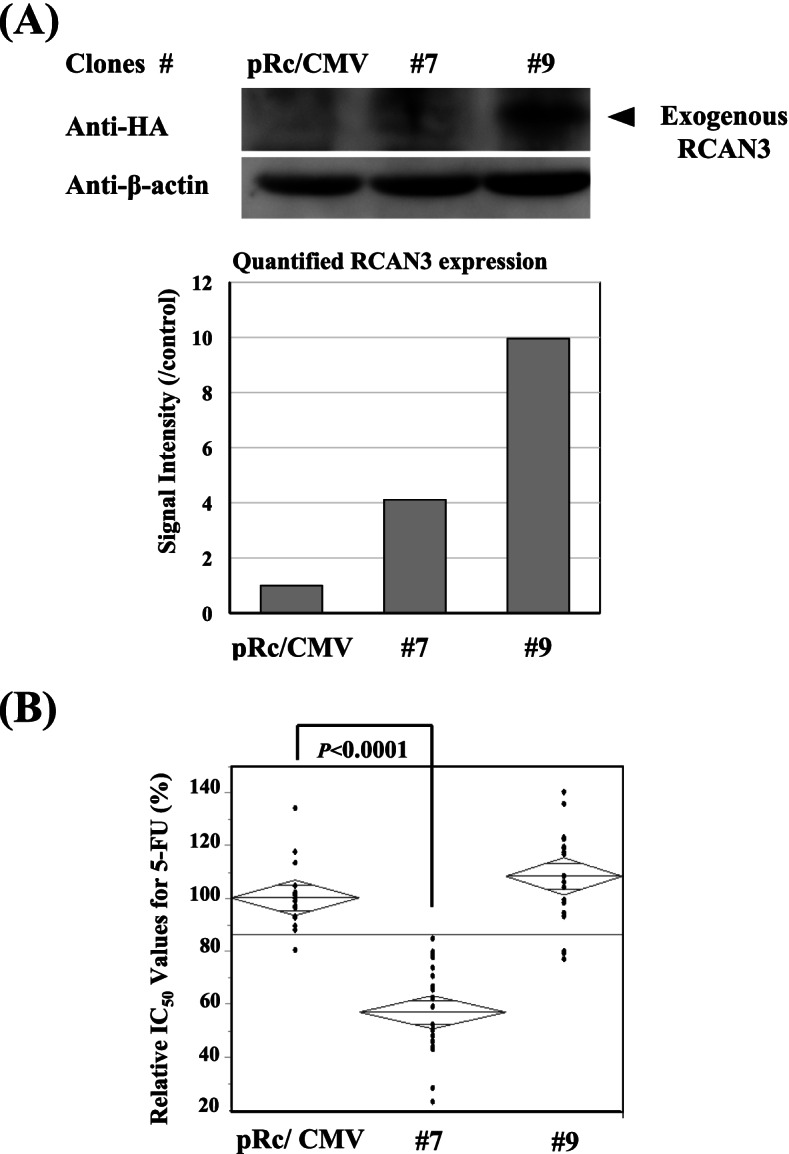
RCAN3 overexpression and cellular 5-FU sensitivity. *RCAN3* transfection into FaDu cells provided 2 stable transfectants with having different levels of RCAN3 expression (**A**). The expected association between RCAN3 expression level and 5-FU sensitivity, however, was not confirmed, differed from the results for AGR2, PDE4D, NINJ2 and CDC25B: The increase of 5-FU sensitivity was observed in the transfectant #7, but such increase was not confirmed at all in the transfectant #9 having the higher expression of RCAN3 (**B**). Western blot analyses and quantification assay on the blots were performed for the target protein and β-actin (**A**) (See Supplemental Information File 1_Western Blot Raw Data)

Although sufficient repression of expression could not be obtained in knock-down analyses, we enabled to establish stable transformants of UT-SCC-26B cells harbouring expression vector of *CDC25B* driven under CMV promoter. These stable transfectants demonstrated that enhanced CDC25B protein expression increased IC_50_ values of 5-FU and attenuated their 5-FU sensitivities (Fig. 6A, B). Interestingly, we also found that the CDC25B overexpression shortened the doubling times of UT-SCC-26B cells (Fig. 6C). CDC25 may play an important role in both cell progression and 5-FU action, and thus can be a potent predictor of cellular 5-FU sensitivity. Despite of little influence on doubling times, transfection analyses of *RCAN3* into FaDu cells demonstrated that overexpression of the gene sensitized their 5-FU sensitivity (Fig. 7). Nevertheless, the expression levels did not correlate with the observed sensitivity to 5-FU in the transfectants: The transfectant #7 having moderately overexpressed RCAN3 were significantly sensitized to 5-FU, while none of the significant change in 5-FU sensitivity was observed in the transfectant #9 having the highest RCAN 3 overexpression.

In summary, knock-down and transfection analyses indicated that high expressions of AGR2 and PDE4D could signify cellular resistance to TXT, while those of NINJ2 and CDC25B might be an indicator of cellular high susceptibility respectively to CDDP and 5-FU.

## Discussion

Biomolecular markers are of considerable value to implement a better or best treatment decision in hypopharyngeal cancers [8, 9, 15, 17]. In this study, we ventured to focus on the conventional key cytotoxic drugs still widely used in practice and newly proposed 4 genes (or proteins) as the most powerful exploratory markers for drug response prediction: They are AGR2 and PDE4D for TXT; NINJ2 for CDDP; and CDC25B for 5-FU. These 4 markers were identified as the most correlative genes (and/or proteins) with cellular sensitivity to TXT, CDDP and 5-FU in the expression levels, through 3-step correlation studies using 15 hypopharyngeal cancer cell lines. The first genome-wide screening study derived 16 highly correlative genes, the second confirmation study using their quantified gene expression data narrowed down the first candidates to 10 genes, and the third study, a series of knock-down and transfection analyses finally provided these 4 molecules as a most potent predictive biomarker. These suggested that the proposed markers would be available correspondingly to both intertumoral heterogeneity and intratumor biological complexity. Despite their action mechanisms are little known, IPA using a large-scale database (TCGA) indicated that each molecule might interacted with main action pathway (and/or target genes) of the relevant drug. These new findings may lead to an assumption that these selected genes could be also novel therapeutic targets with unique action mechanisms. Up to the present, a considerable number of candidates have been proposed as a predictive marker of therapeutic response for the taxane, platinum or 5-FU chemotherapy, though they are still controversial [34–37]. This is the first report to exploit the latent roles of these 4 molecules in current standard cancer chemotherapy for hypopharyngeal cancer patients, we believe. Our findings could afford a key to the development of precision medicine for hypopharyngeal cancers.

Plenty of studies have demonstrated the involvement of all 4 molecules in many cancers other than hypopharyngeal cancer. AGR2, an endoplasmic reticulum (ER)-resident protein disulfide isomerase (PDI), has been generating a massive interest because of its overexpression and vital roles in various cancers [37, 38]. The ER-resident PDI is essential in the production of gel-forming mucins to protect cells from ER stress and maintains ER homeostasis. AGR2 is known to be upregulated in various epithelial tumors, which promotes aggressive growth, survival, angiogenesis, senescence, metastasis, and drug resistance of tumors through several pathways including p53 activation pathway. The potential as a predictive biomarker of therapeutic response has been shown in various types of drugs: cytotoxic agents including TXT, angiogenesis inhibitors, hormonal therapeutic agents, molecular targeted drugs, and immune checkpoint inhibitors, but also the clinical significance along with its functional mechanism are still undetermined [39].

PDE4D, a subtype of an isoform of type 4 cyclic nucleotides phosphodiesterases, is involved in regulation of cyclic adenosine monophosphate (cAMP) and plays a major role in cellular signaling and thus cell proliferation [40]. The PDE4 subtype recently appeared to be associated with several cancers including central nerve system tumors, hematologic malignancies and several solid cancers, and the upregulation are suggested to participate in cancer chemoresistance [41].

In contrast to the 3 drug resistant predictors, the other selected genes (or protein), namely-NINJ2 for CDDP, and CDC25B for 5-FU, appeared to work as a predictor of cellular susceptibility to the relevant drugs. The higher their expressions cells have, the more cells are susceptible to the relevant drug, we found. Nonetheless, there was a little to what we could learn from the literature study on NINJ2: NINJ2 is a cell surface adhesion molecule upregulated in neurons and grail cells and several cancers [42]. Some reports have suggested that NINJ2 could work as an oncogenic protein and the overexpression promotes cell growth in glioma and colorectal cancer cells [43, 44]. The biological roles in cancer including the involvement in cellular chemosensitivity are very little known. Interestingly, a study on NINJ1, a homologue of NINJ2, have suggested that NINJ1 exerts two opposing effects on cell growth, migration, and tumorigenesis via WT and mutant p53, with the findings that cell proliferation and migration can be inhibited by the loss of NINJ1 in cells carrying WT p53 [44]. More recently, cell-surface NINJ1 protein has an essential role in the induction of plasma membrane rupture, the final cataclysmic event in lytic cell death [45]. These might explain somewhat our results that NINJ2 overexpressed hypopharyngeal cancer cells were more sensitive to CDDP. CDDP induces cytotoxic activity through its interference in DNA replication by eliciting drug-DNA cross links and we found that NINJ2 might connect with DNA replication pathway. These encouraged us to line up NINJ2 as a candidate of putative predictive marker for CDDP for further investigation.

CDC25B is a key player in G2/M cell cycle progression and well known to be upregulated in various cancers [46], but the specific mechanisms of CDC25B in tumor progression is still uncertain as with the other 3 molecules. Various reports demonstrated that the overexpression has an oncogenic property cooperated with either oncogenic HRAS or Rb inactivation and is attributed also to a target of both oncogene c-myc and tumor suppressor p53 [47]. Contrary to the findings, recent articles revealed that CDC25B induces cellular senescence and correlates tumor suppression in a p53-dependent manner [48]. Extensive efforts have been focused also on the roles of CDC25B in drug resistance. Among CDC25B-mediated cell cycle slowdown is likely a most probable mechanism since the phosphatase acts on resuming the cell cycle after the arrest: 5-FU resistance is associated with a significant delay of cell cycle in G_1_ and G_1_/S and prolonged DNA synthesis time, which prevents incorporation of 5-FU metabolite into DNA. The mechanism is shown to be mediated by the activation of G_2_M checkpoint kinase 2 (CHK2) and the subsequent decrease in CDC25B expression [49]. This putative mechanism may well match with our findings. In this study, IPA revealed that CDC25B might interact with DNA synthesis pathway and TYMS, main action pathway and drug target of 5-FU. CDC25B expression levels were inversely correlated of with cellular 5-FU resistance. CDC25B up-regulation shortened doubling times of UT-SCC-26B cells in association with sensitization of the cells to -FU. Cell cycle slowdown mechanism may be of key importance in CDC25B-mediated 5-FU resistance.

Drug resistant determinants can be an attractive therapeutic target to improve outcome of cancer patients. As shown in our knock-down experiments of AGR2, depletion or repression of such determinants can conquer cellular resistance to the relevant drug. AGR2 and PDE4D might be not only a distinctive predictive marker of TXT resistance but also a relevant therapeutic target to overcome the resistance. In contrast, induction of NINJ2 and CDC25 would be a powerful way to enhance therapeutic efficacy of CDDP and 5-FU respectively, since negative expression of them signified cellular resistance to the drugs. The potential of these 4 molecules as a novel drug target also has been shown in a variety of studies: An accumulated knowledge on AGR2, PDE4D, NINJ2 and CDC25B have accelerated the drug discovery targeting these molecules in the other cancers. Despite of the extensive efforts, none of the clinically available target drugs, however, have been developed yet [50–54]. The research on drug discovery targeting the 4 molecules remain rudimentary.

Our findings raise the great potential of 4 molecules to be not only a predictive biomarker but also a therapeutic target in hypopharyngeal cancer, but the limited knowledge of their biological roles in cancer become a big obstacle to the development of novel therapeutics. Even their roles in cancer biology including oncogenesis and tumor progression are still under investigation. Elucidation of a role of selected candidate in the pathophysiology of a disease and/or disease-modifying is mandatory required in the process of a promising target identification for drug discovery. The relevance of STXBP4 to AGR2 also is unknown. The more detailed biological functions of selected 4 molecules need to be elucidated. Along with continuing our basic research in more depth, we should conduct research to identify possible molecular targeting drugs for the 4 molecules and their best therapeutic composition based on the understanding.

We suggested the close connection of 4 marker candidates with main action pathway (or targets) of the relevant drug, TXT, CDDP or 5-FU, but the detailed mechanisms are also unidentified. It is necessary also to show the significance of all 4 molecules as a cellular drug sensitivity (or resistance) modifier more clearly. Despite of reasons unknown, several knock-down and gene transfection analyses did not work well enough to evaluate the correlation of target genes with dug sensitivity in the expression level. There observed some discrepant results between gene and protein expression in the gene knock-down and transfection variants. These could be explained due to the limited number of current experiments, such as in the Western blot analyses, and/or some technical errors at least in a part but might suggest the existence of some intricate mechanisms underlying the phenomenon.

We are now planning both in vitro and in vivo research to address them. As the first step, we intend to elucidate the functional roles of the 4 molecules in cancer biology in the similar manner as we did to clarify the essential role of STXBP4 [26].

## Conclusions

Herein, we newly demonstrated that 4 genes (and the proteins) as the most powerful exploratory markers for response prediction to TXT, CDDP, and 5-FU- namely, AGR2, and PDE4D for TXT; NINJ2 and possibly RAB15 for CDDP, and CDC25B for 5-FU- in hypopharyngeal cancers. Their relevance to cancer biology, however, is little known and hypopharyngeal cancer is relatively rare cancer with great heterogeneity. Although the way to the goal will be a tough challenge, the findings raise the potential not only to realize a precision medicine in the essential treatment but also lead to the development of novel molecular targeting drugs.

## Supplementary Information

**Additional file 1: Supplementary File 1.** Primers and Universal Probe Library probes used for the real-time RT-PCR analysis

**Additional file 2: Supplementary File 2.** Primers used for the construction of plasmid

**Additional file 3: Supplementary Dataset File 1.** Correlation analysis data between microarray gene expression data and cellular docetaxel (TXT) sensitivity (IC50 value)

**Additional file 4: Supplementary Dataset File 2.** Correlation analysis data between microarray gene expression data and cellular cis-platinum (CDDP) sensitivity (IC50 value)

**Additional file 5: Supplementary Dataset File 3.** Correlation analysis data between microarray gene expression data and cellular 5-fluorouracil (5-FU) sensitivity (IC50 value)

**Additional file 6: Supplementary Dataset File 4.** Functional analysis data of AGR2, PDE4D, and RAB15 selected as potent docetaxel (TXT) sensitivity markers.

**Additional file 7: Supplementary Dataset File 5.** Functional analysis data of KLK11, NINJ2, and PTGS1, selected as potent cis-platinum (CDDP) sensitivity markers.

**Additional file 8: Supplementary Dataset File 6.** Functional analysis data of KLK11, NINJ2, and PTGS1 selected as potent 5-fluorouracil (5-FU) sensitivity markers.

**Additional file 9: Supplementary Information File 1_Wester Blot Raw Data.** Western Blot Analysis: Full-length gels and blots for (1) AGR2 and β-actin, ( cf. Figure 2 (C)), (2) PDE4D and β-actin (cf. Figure 3(A)), (3) RAB15 and β-actin (cf. Figure 4 (A)), (4) CDC25B and β-actin (cf. Figure 6 (A)), and (5) RCAN3 and β-actin (cf. Figure 7 (A))

## Data Availability

The data of this study were derived from the The Cancer Genome Atlas (TCGA) and Human Protein Atlas, which were available respectively from: https://www.cancer.gov/about-nci/organization/ccg/research/structural-genomics/tcga and https://www.proteinatlas.org/. The datasets used during the current study are available from the Supplementary Files, Supplementary Dataset Files, Supplementary Information Files and the corresponding author on reasonable request.

## Abbreviations

TXT: docetaxel
CDDP: cis-platinum
5-FU: 5-fluorouracil
SCC: squamous cell carcinoma MTT [3-(4,5-dimethylthiazol-2-yl)-2,5-diphenyltetrazolium bromide]
FBS: fetal bovine serum
IC_50_: inhibitory drug concentration of 50% cell growth or drug concentration of 50% optical density of control
PCR: polymerase chain reaction
ACTB: actin β
GAPDH: glyceraldehyde-3-phosphate dehydrogenase
AGR2: anterior gradient 2 homolog
NINJ2: ninjurin 2
siRNA: small interfering RNA
PDE4D: phosphodiesterase 4D cAMP-specific
RAB15: member RAS onocogene family
CDC25B: cell division cycle 25 homolog B
RCAN3: RCAN family member 3
IPA: Ingenuity Pathway Analysis
SYNGR1: synaptogyrin 1
KLK11: kallikrein-related peptidase 11
PTGS1: prostaglandin-endoperoxide synthase 1
PBX3: pre-B-cell leukemia homeobox 3
SEPW1 (SELENOW): selenoprotein W, 1
TUBB: tublin β
TYMS: thymidylate synthetase
KRAS: v-Ki-ras2 Kirsten rat sarcoma viral oncogene homolog
TP53: tumor protein 53
OBSL1: obscurin-like 1 gene
STXBP4: syntaxin binding protein 4
ΔNp63: (an isoform of tumor protein 63)
TP63: tumor protein 63
ER: endoplasmic reticulum
PDI: protein disulfide isomerase
cAMP: cyclic adenosine monophosphate
circRNA: circular RNA expression
CN-NFATc: the calcineurin-cytosolic nuclear factor of activated T cells
MTT: [3-(4,5-dimethylthiazol-2-yl)-2,5-diphenyltetrazolium bromide]
