# Supplementary material for: Biomarker discovery for practice of precision medicine in hypopharyngeal cancer: a theranostic study on response prediction of the key therapeutic agents
Source: BMC Cancer. 2022 Jul 16;22:779. doi: 10.1186/s12885-022-09853-1 (PMC9288037; doi:10.1186/s12885-022-09853-1)
Supplement: Supplementary file 1 — Additional file 1: Supplementary File 1. Primers and Universal Probe Library probes used for the real-time RT-PCR analysis [file 12885_2022_9853_MOESM1_ESM.docx]

**Supplementary File 1. Primers and Universal Probe Library probes used for the real-time RT-PCR analysis**

| Drug | Gene symbol | Forward Primer | Reverse Primer |
| --- | --- | --- | --- |
| TXT | *AGR2* | GGTGGGTGAGGAAATCCAG | GTAGGAGAGGCCACAAGG |
|  | *SYNGR1* | TCGCCTTCTCCTTTTTCTCC | ARCTGGTACCGCTGGAAGG |
|  | *PDE4D* | TTGTCCAGTCTACTCATGTGCTATT | TTGCTGCAAGAATCTCCAAA |
|  | *RAB15* | GGCTGATGAGGAGCAGAAAC | TCCATGCCATACTCCTTGG |
| CDDP | *NOTCH2NL* | GGAGGCGACCGAGAAGATA | GCCATCTCGACACTGCAA |
|  | *KLK11* | GCTTGCTCTGGCAACAGG | AGTGAGGCTTGCACTCGAAC |
|  | *NINJ2* | CCCTGGTCACCCTCATCA | CGCCACTGCTTTTCTACCTC |
|  | *PTGS1* | TTCTCTCGCCAGATTGCTG | CCGAGACTCCCTGATGACA |
| 5-FU | *GOLGA8A* | GGTGGTTGCCACTCATCTG | GGACTGCTGCTTGTTCTTGG |
|  | *JDP2* | AAGGAGCGCACGGAGTTT | ATCTGGGTCTTCAGCTCTGC |
|  | *STXBP1* | GCCTGGAGGCTGTGTGTATCTC | CGGGTCTTAAAGTCACTGATG |
|  | *CDC25B* | TGCAGGTCTCTGCATGGAT | GGATGGCCTGTTCAAACG |
|  | *PBX3* | AGCGTCCTGTGTGTGAGATCAA | GTCTCATTAGCTGGGGATCG |
|  | *SEPW1* | CTTGGCTCAGGGCTAATGC | CCTATCATGAAGCGTCTGCTG |
|  | *RCAN3* | CGCGAATAGAACTCCACGA | GACTTGTCCCGCACTTGG |
|  | *ZNF584* | GGGCCTATACCGGGATGT | GATCTCGAAGGTGCAAGTCC |
| Control | *HPRT1* | TGACCTTGATTTATTTTGCATACC | CGAGCAAGACGTTCAGTCCT |
