# Supplementary material for: Biomarker discovery for practice of precision medicine in hypopharyngeal cancer: a theranostic study on response prediction of the key therapeutic agents
Source: BMC Cancer. 2022 Jul 16;22:779. doi: 10.1186/s12885-022-09853-1 (PMC9288037; doi:10.1186/s12885-022-09853-1)
Supplement: Supplementary file 2 — Additional file 2: Supplementary File 2. Primers used for the construction of plasmid [file 12885_2022_9853_MOESM2_ESM.docx]

**Supplementary File 2. Primers used for the construction of plasmid**

| Gene symbol | Forward Primer | Reverse Primer |
| --- | --- | --- |
| *AGR2* | GCCGCCATGGAGAAAATTCCAGTGTCAGC | ACGTTCTAGACAATTCAGTCTTCAGCAACTTGAGAG |
| *PDE4D* | GCCGCCATGGAGGCAGAGGGCAGC | ACGTTCTAGACGTGTCAGGAGAACGATCATCTATG |
| *RAB15* | GCCGCCATGGCGAAGCAGTACGATGTG | ACGTTCTAGAGACGCGTGAATGACTCTTTAATGTTG |
| *CDC25B* | GCCGCCATGGAGGTGCCCCAGCCG | ACGTTCTAGACTGGTCCTGCAGCCGGCTAC |
| *RCAN3* | GCCGCCATGCTGAGGGACACTATGAAATCTTGG | AGCTTCTAGACAGCGCGCAATCAAAGGTCT |
