# Supplementary material for: Biomarker discovery for practice of precision medicine in hypopharyngeal cancer: a theranostic study on response prediction of the key therapeutic agents
Source: BMC Cancer. 2022 Jul 16;22:779. doi: 10.1186/s12885-022-09853-1 (PMC9288037; doi:10.1186/s12885-022-09853-1)
Supplement: Supplementary file 9 — Additional file 9: Supplementary Information File 1_Wester Blot Raw Data. Western Blot Analysis: Full-length gels and blots for (1) AGR2 and β-actin, ( cf. Figure 2 (C)), (2) PDE4D and β-actin (cf. Figure 3(A)), (3) RAB15 and β-actin (cf. Figure 4 (A)), (4) CDC25B and β-actin (cf. Figure 6 (A)), and (5) RCAN3 and β-actin (cf. Figure 7 (A)) [file 12885_2022_9853_MOESM9_ESM.pdf]

Cf. Figure 2 (C) AGR2 expresson

Cells: UMB-SCC-745 cell

Vector: pRc/CMV (Cont) and pRc/CMV + *AGR2* (#1, #6)

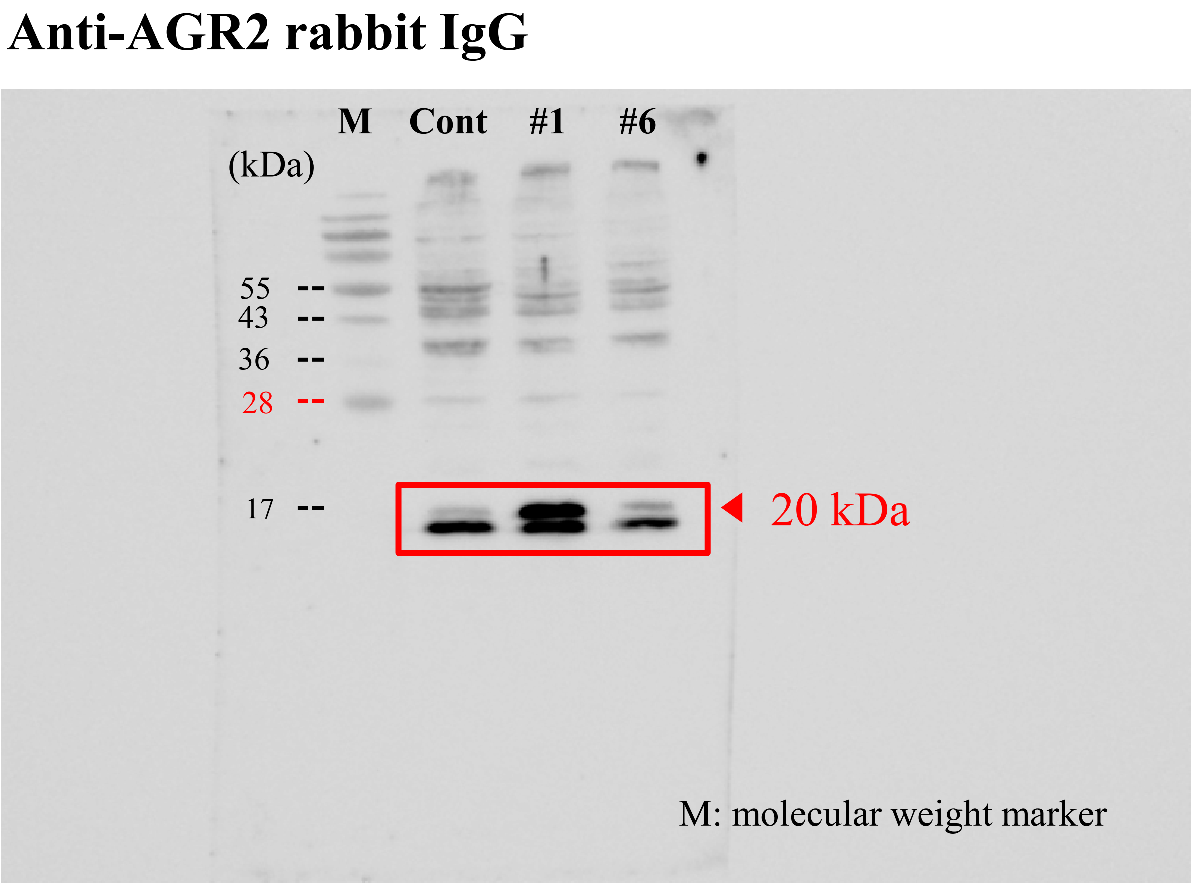

Primary antibody : Abcam anti-AGR2 rabbit IgG (1:10000)
Secondary antibody : Abcam anti-rabbit IgG-HRP (1:140000)

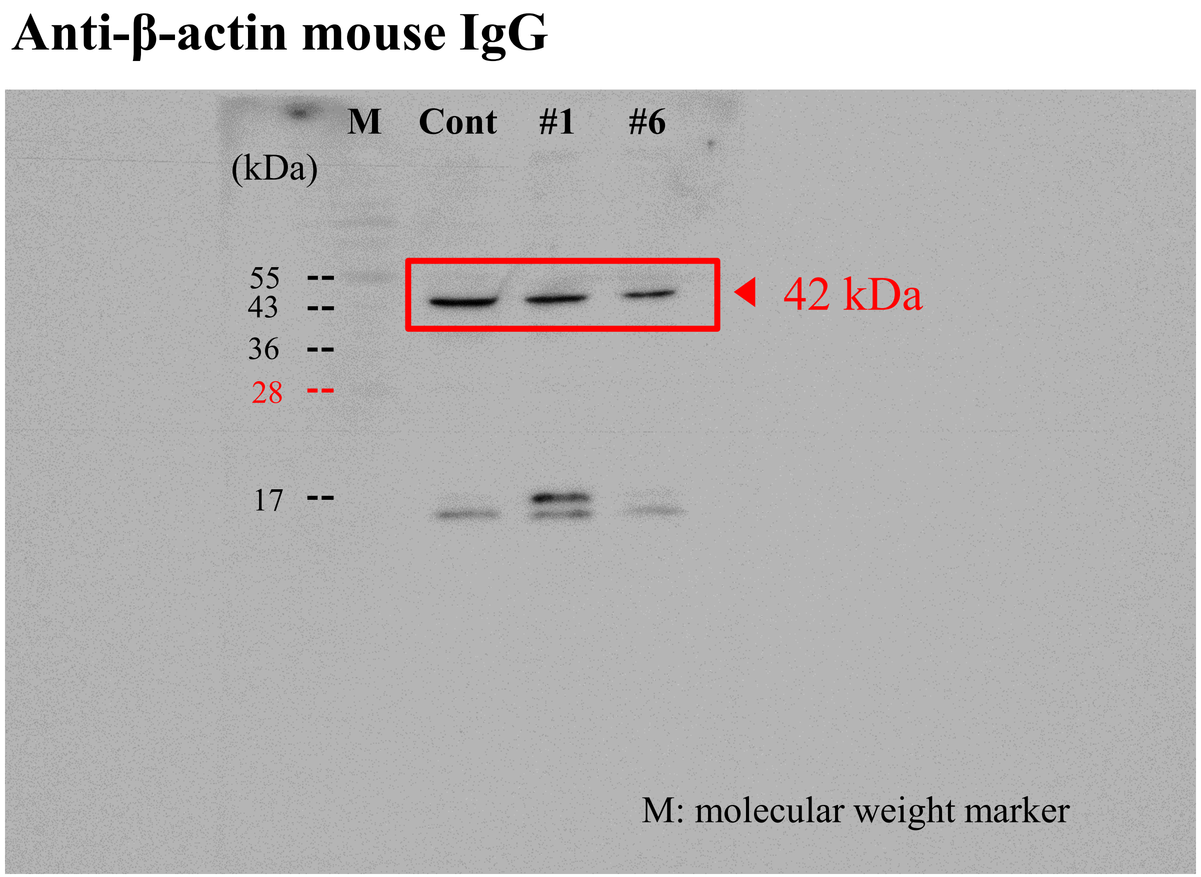

Primary antibody : Abcam anti-β-actin mouse IgG (1:5000)
Secondary antibody : GE anti-mouse IgG-HRP (1:10000)
(Using 1 membrane without re-probing)

Cf. Figure 3 (A) RAB15 expression

Cells: BICR6 cell

Vector: pRc/CMV (Cont) and pRc/CMV + *RAB15* (#7, #10)

Anti-HA rat IgG

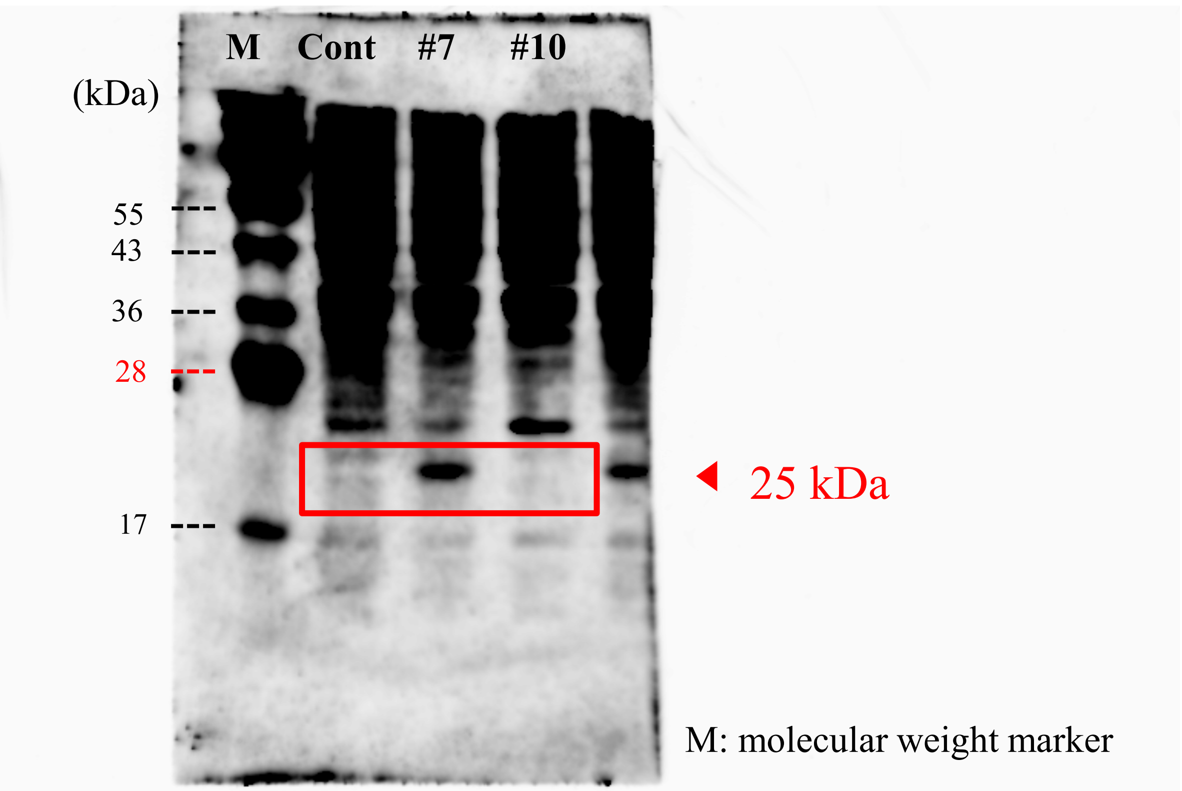

Primary antibody : Roche anti-HA rat IgG (1:1000)
Secondary antibody : GE anti-rat IgG-HRP (1:10000)

Anti-β-actin mouse IgG

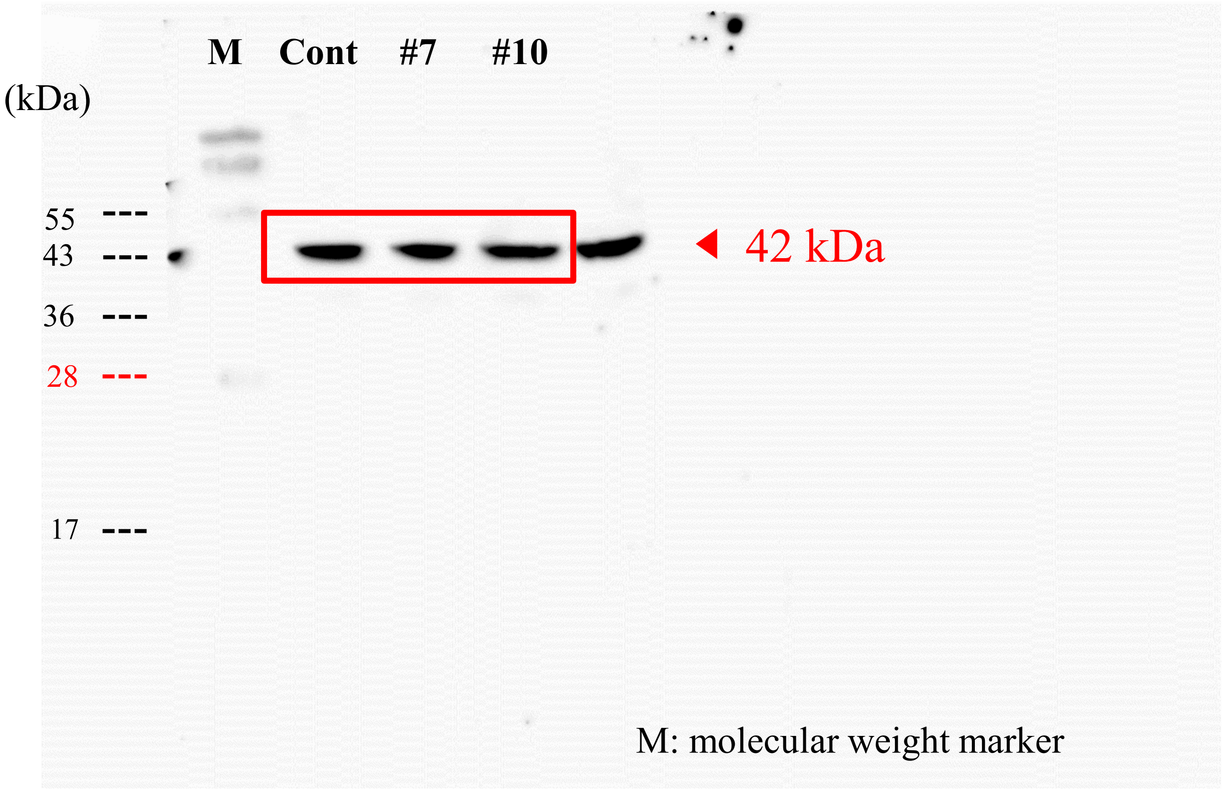

Primary antibody : Abcam anti-β-actin mouse IgG (1:5000)
Secondary antibody : GE anti-mouse IgG-HRP (1:10000)
(Using another membrane transferred from the same electrophoresed gel)

cf. Figure 4 (A) PDE4D expression

Cells: BICR6 cell

Vector: pRc/CMV (Cont) and pRc/CMV + *PDE4D* (#6, #8, #9, #10)

Anti-HA rat IgG

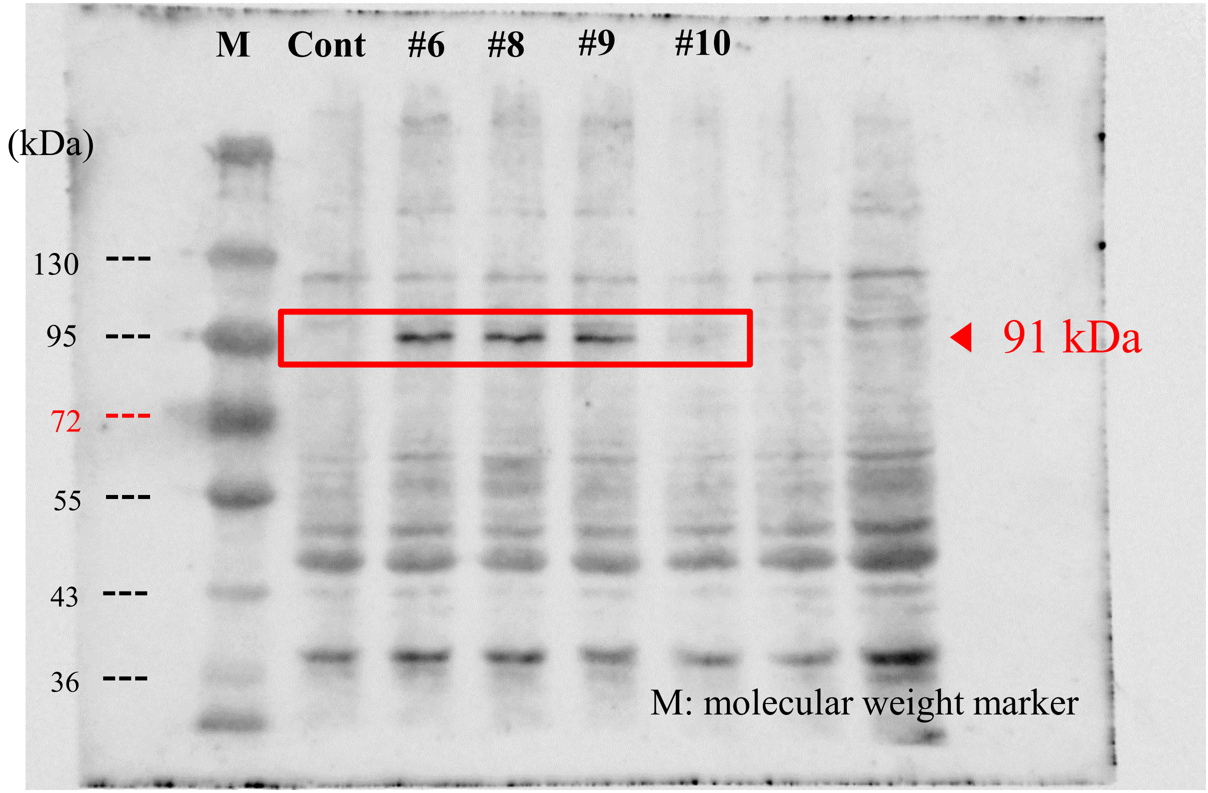

Primary antibody : Roche anti-HA rat IgG (1:2000)
Secondary antibody : GE anti-rat IgG-HRP (1:10000)

Anti-β-actin mouse IgG

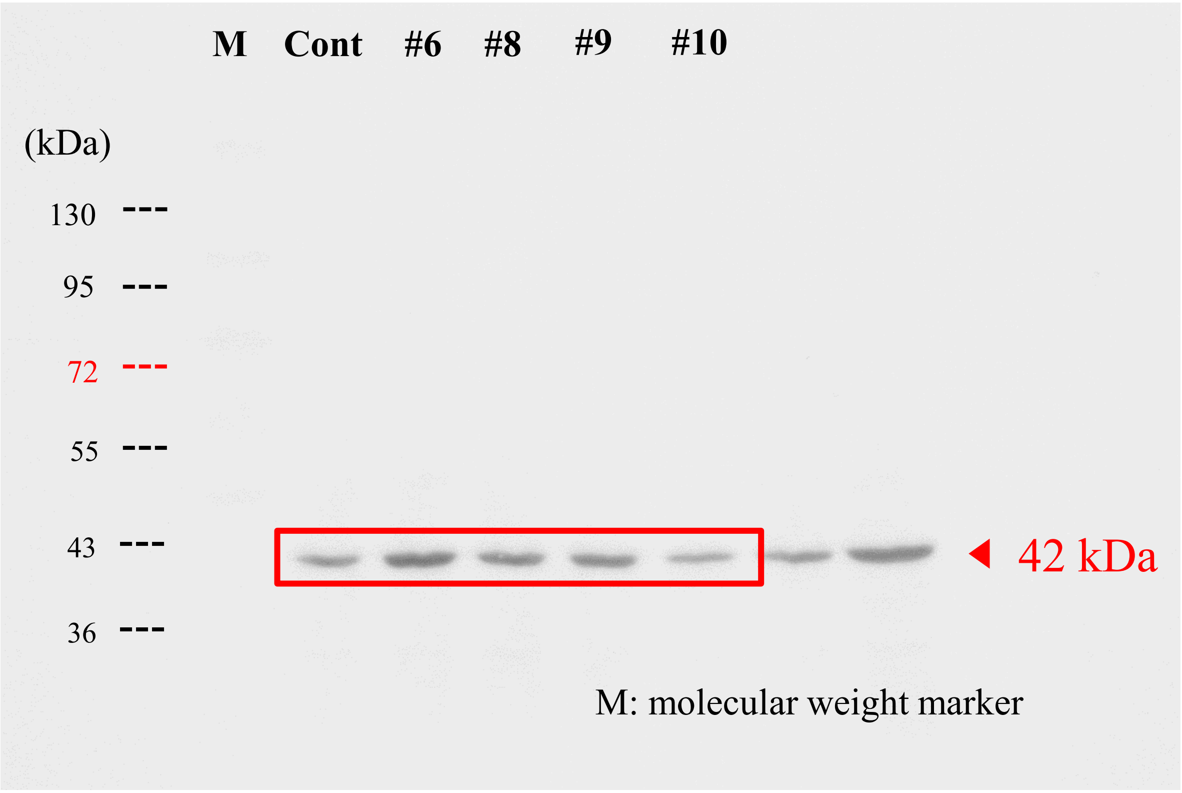

Primary antibody : Abcam anti-β-actin mouse IgG (1:5000)
Secondary antibody : GE anti-mouse IgG-HRP (1:10000)
(Reusing the same membrane for the subsequent antibody probes)

cf. Figure 6 (A) CDC25B expression

Cells: UT-SCC-26B cell

Vector: pRc/CMV (Cont) and pRc/CMV + *CDC25B* (#5, #6, #12, #14)

Anti-HA rat IgG

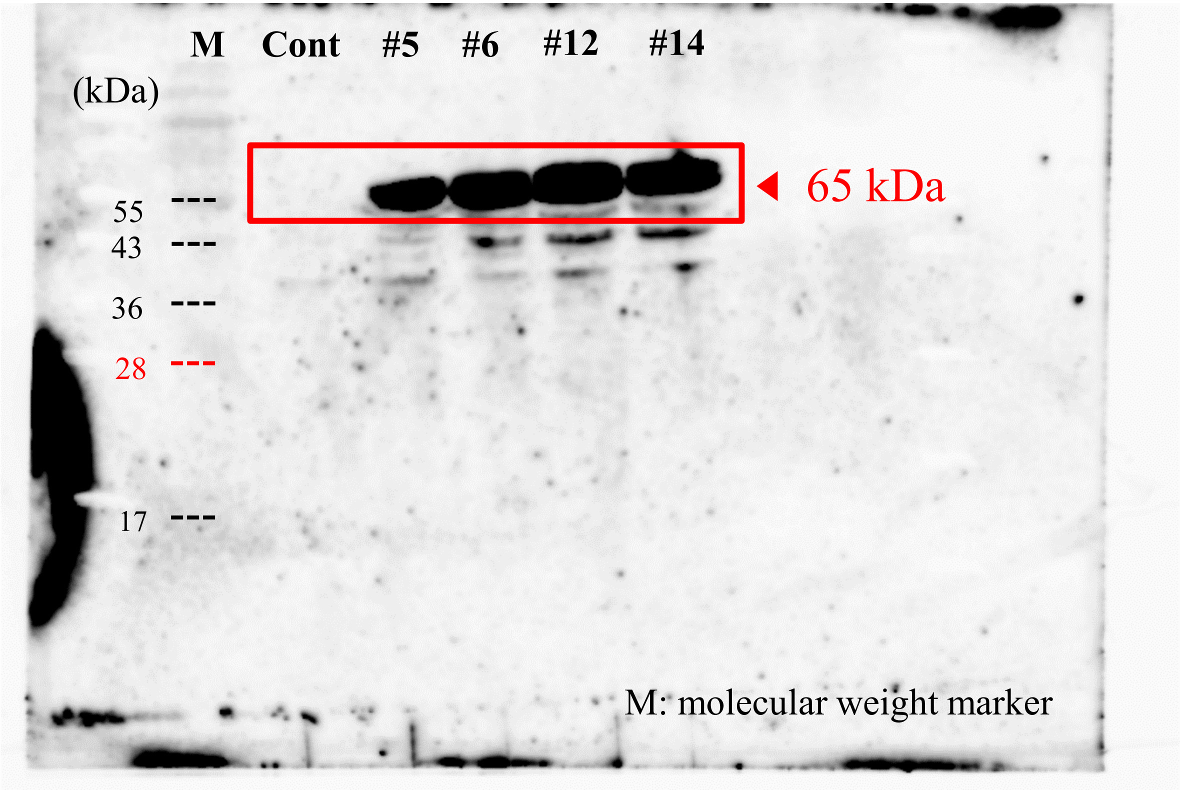

Primary antibody : Roche anti-HA rat IgG (1:1000)
Secondary antibody : GE anti-rat IgG-HRP (1:10000)

Anti-β-actin mouse IgG

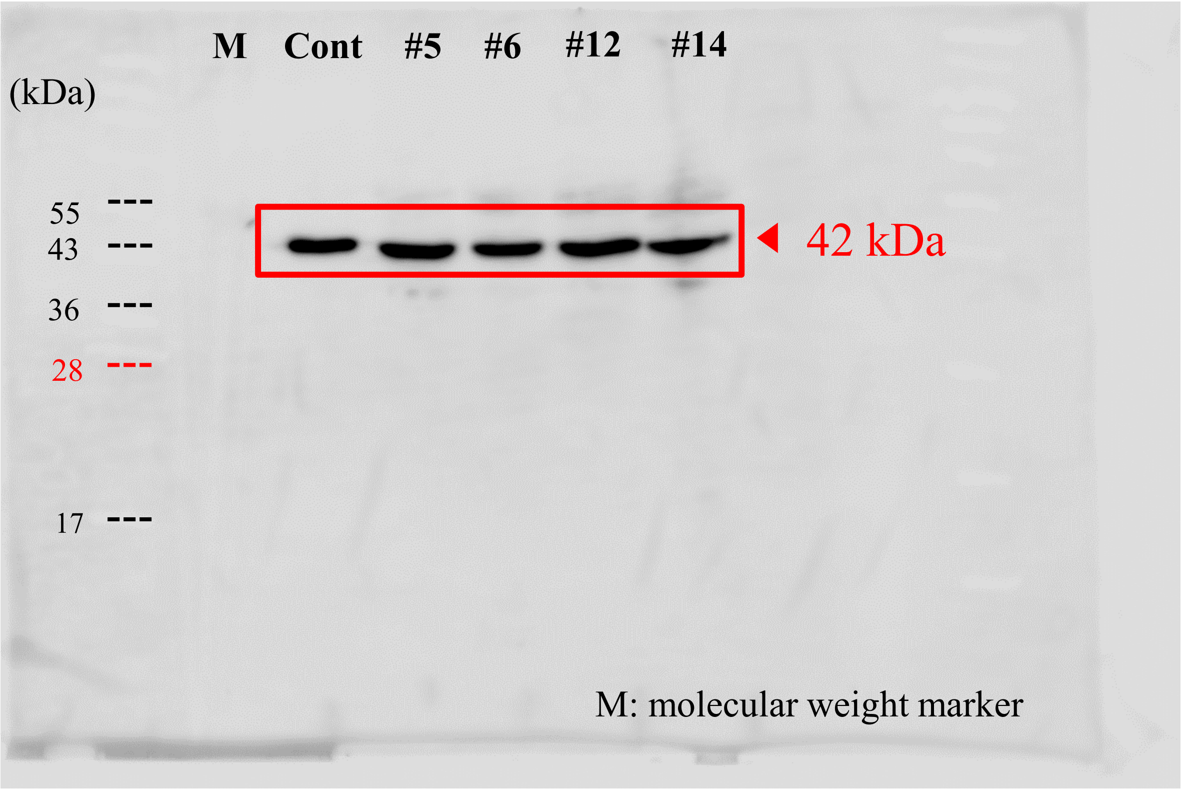

Primary antibody : Abcam anti-β-actin mouse IgG (1:5000)
Secondary antibody : GE anti-mouse IgG-HRP (1:10000)
(Reusing the same membrane for the subsequent antibody probes)

Cf. Figure 7 (A) RCAN3 expression

Cells: FaDu cell

Vector: pRc/CMV (Cont) and pRc/CMV + *RCAN3* (#7, #9)

Anti-HA rat IgG

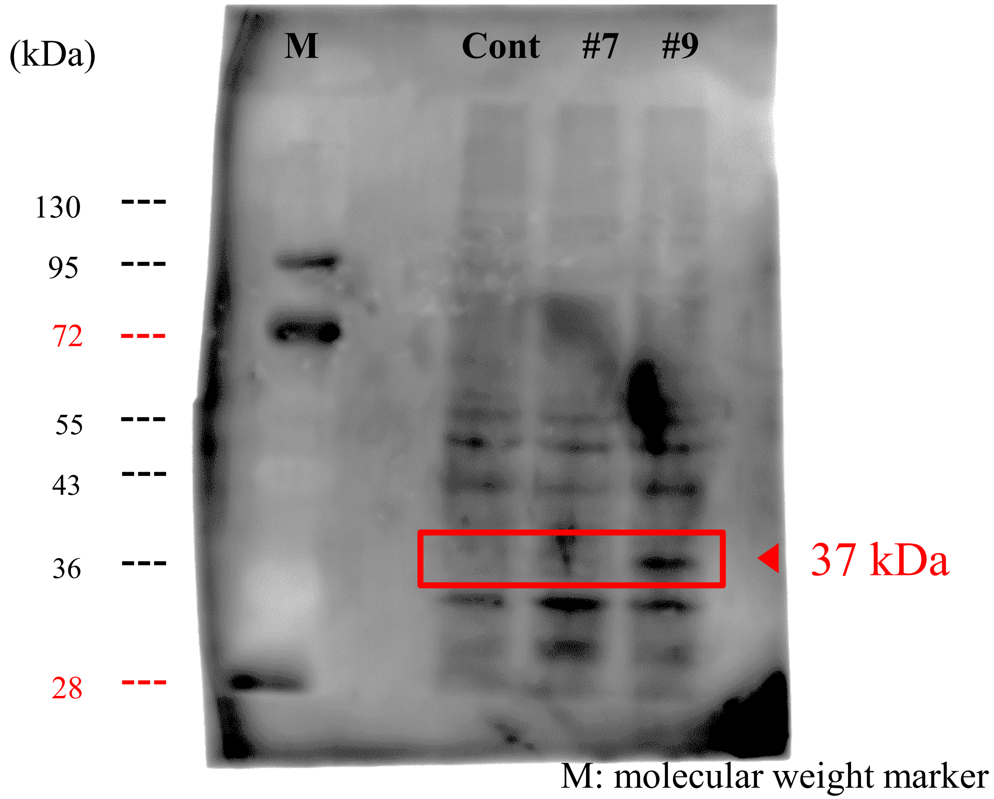

Primary antibody : Roche anti-HA rat IgG (1:1000)
Secondary antibody : Abcam anti-rat IgG-HRP (1:10000)

Anti-β-actin mouse IgG

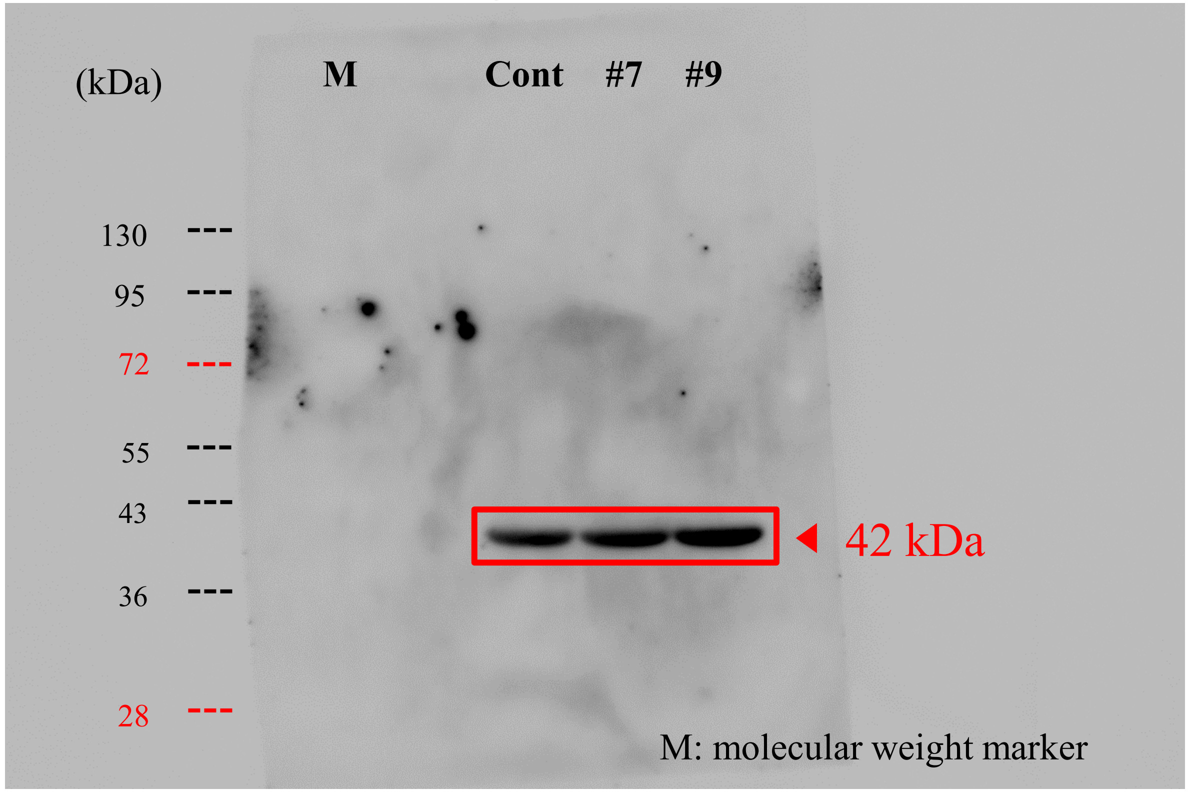

Primary antibody : Abcam anti-β-actin mouse IgG (1:5000)
Secondary antibody : GE anti-mouse IgG-HRP (1:10000)
(Reusing the same membrane for the subsequent antibody probes)
